# Supplementary material for: Content-rich biological network constructed by mining PubMed abstracts
Source: BMC Bioinformatics. 2004 Oct 8;5:147. doi: 10.1186/1471-2105-5-147 (PMC528731; doi:10.1186/1471-2105-5-147)
Supplement: Additional File 2 — The original results of the above study (non-essential files are deleted to keep the file size under the limit set by BMC bioinformatics). [file 1471-2105-5-147-S2.bz2 › chilibotAdditionalFile2/dip40/34ID7566124E118/html/CASP3_GZMB.html]

 


 **CASP3** and **GZMB** 
  
Found 75 abstracts in PubMed, retrieved 40.  
 

 What does Google say? 
 PDF only 
| .edu only 

---

**Interactive relationship** (e.g. stimulation, inhibition, etc)

**Inhibitory relationship**- Thus granzyme B  [ **GZMB** ]  mediates direct cleavage of caspase 3  [ **CASP3** ]  and also activates mitochondrial disruption, resulting in the release of proapoptotic proteins that suppress caspase inhibition.  Ref: 12648453 Immunity, 2003
- The LAK cell enhanced caspase 3  [ **CASP3** ]  activity in the pretreated OSC 3 cells was decreased to approximately 70% and 40% of the control by the addition of Z AAD CMK a granzyme B  [ **GZMB** ]  inhibitor and neutralising monoclonal antibody to Fas antigen alphaFas IgG, respectively.  Ref: 11000584 Eur J Cancer, 2000
**Neutral relationship**- The apoptotic pathway distal to the DISC is intact because ceramide analogs, staurosporine, and granzyme B  [ **GZMB** ]  activate caspase 3  [ **CASP3** ]  and induce apoptosis.  Ref: 10477698 Blood, 1999
- Once in the cytosol, granzyme B  [ **GZMB** ]  targets caspase 3  [ **CASP3** ]  directly or indirectly through the mitochondria, initiating the caspase cascade to DNA fragmentation and apoptosis.  Ref: 12752668 Immunol Rev, 2003
- The three dimensional structure of human granzyme B  [ **GZMB** ]  compared to caspase 3  [ **CASP3** ] , key mediators of cell death with cleavage specificity for aspartic acid in P1.  Ref: 11325591 Chem Biol, 2001
- Caspase 3  [ **CASP3** ]  activation occurred in CC531s cells after perforin granzyme B  [ **GZMB** ]  treatment, but not after the addition of recombinant FasL.  Ref: 11459173 Cancer Immunol Immunother, 2001
- Recent work shows that granzyme B  [ **GZMB** ]  mediated release of apoptotic factors from the mitochondria is essential for the full activation of caspase 3  [ **CASP3** ] .  Ref: 12752668 Immunol Rev, 2003
- Strikingly, only the DEVD cleaving caspases, caspase 3  [ **CASP3** ]  and caspase 7, inactivated DFF45 ICAD and promoted DNA fragmentation in an in vitro DFF40 CAD assay, suggesting that granzyme B  [ **GZMB** ] , caspase 6, and caspase 8 promote DFF45 ICAD inactivation and DNA fragmentation indirectly by activating caspase 3  [ **CASP3** ]  and or caspase 7.  Ref: 10521451 J Biol Chem, 1999
- Cultured renal epithelial MDCK C7 and MDCK C11  [ **GZMB** ]  or OK cells also showed increased caspase 3  [ **CASP3** ]  activity after OTA exposure.  Ref: 10811536 Cell Biol Toxicol, 1999

**Non-interactive relationship** (e.g. studied together, co-existance, homology, etc.)

- These results indicate that anticancer drugs and gamma rays prime squamous cell carcinoma cells to be susceptible to apoptosis by LAK cells, that LAK cell induced apoptosis largely depends on the activation of caspase 3  [ **CASP3** ]  by the Fas Fas ligand signal and granzyme B  [ **GZMB** ] ,.  Ref: 11000584 Eur J Cancer, 2000
- The DS brain was examined using immunocytochemistry and antibodies against the active fragment of caspase 8 AC, 8 and to caspase 3  [ **CASP3** ]  cleavage products of fodrin CCP  [ **GZMB** ] , a neuronal cytoskeleton protein.  Ref: 12213643 Neurosci Lett, 2002
- The aims of this study were to determine whether CC cells are also resistant to perforin granzyme B  [ **GZMB** ]  and whether the FasL resistance lies upstream of caspase 3  [ **CASP3** ]  activation.  Ref: 11459173 Cancer Immunol Immunother, 2001
- The apoptosis associated marker Fas, FasL, caspase 3  [ **CASP3** ]  and granzyme B  [ **GZMB** ]  were expressed by morphologically neoplastic cells in CTCL and by large atypical cells in LyP, with no significant differences.  Ref: 11899146 Br J Dermatol, 2001
- Immunohistochemical demonstration of the expression of th following markers was performed on formalin fixed skin sections Fas, Fas ligand bcl 2, granzyme B  [ **GZMB** ] , the tumour suppressor protein PTEN and the effector caspase, caspase 3  [ **CASP3** ] .  Ref: 11899146 Br J Dermatol, 2001
- Anti beta fodrin Abs in patients sera as well as mouse polyclonal sera raised against the amino terminal beta fodrin fragment did not react with intact beta fodrin, but recognized the 65 kDa amino terminal fragment generated through cleavage by caspase 3  [ **CASP3** ]  or granzyme B  [ **GZMB** ] .  Ref: 11673564 J Immunol, 2001
- Since both the FasL and perforin granzyme B  [ **GZMB** ]  pathways converge at caspase 3  [ **CASP3** ] , we measured caspase 3  [ **CASP3** ]  activity to learn whether the FasL resistance was due to failure to activate this crucial executioner.  Ref: 11459173 Cancer Immunol Immunother, 2001
- Together, these results suggest that CC cells are sensitive to perforin granzyme B  [ **GZMB** ]  induced apoptosis by activating caspase 3  [ **CASP3** ]  and FasL resistance lies upstream of this executioner caspase.  Ref: 11459173 Cancer Immunol Immunother, 2001
- We demonstrated the localization of CD56, perforin, granzyme B  [ **GZMB** ]  and caspase 3  [ **CASP3** ]  in the endometrium by immunohistochemistry.  Ref: 11321047 Tohoku J Exp Med, 2001
- These results provide evidence for a two pronged strategy for mediating target cell destruction and provide evidence of a direct link between granzyme B  [ **GZMB** ]  activity, Bid cleavage, and caspase 3  [ **CASP3** ]  activation in whole cells.  Ref: 10805722 Mol Cell Biol, 2000
- Furthermore, we showed that caspase 3  [ **CASP3** ]  activity is involved in the execution of perforin granzyme B  [ **GZMB** ]  induced apoptosis in CC531 s cells, since the cell permeable caspase 3  [ **CASP3** ]  inhibitor Z DEVD FMK abrogated DNA fragmentation.  Ref: 11459173 Cancer Immunol Immunother, 2001
- In this report, we demonstrate that  **GzmB**  can initiate apoptosis in the absence of caspase 3  [ **CASP3** ]  activity by directly cleaving DFF45 ICAD to liberate activated DFF40 CAD.  Ref: 10894162 Immunity, 2000
- Actual execution of apoptosis depends on proper functioning of effector caspases, particularly caspase 3  [ **CASP3** ] , and on the expression levels of apoptosis regulating proteins, including Bcl 2 and the recently identified granzyme B  [ **GZMB** ]  specific protease inhibitor 9 PI9.  Ref: 12036886 Blood, 2002
- In vitro translated AHNAK fragment could be cleaved by granzyme B  [ **GZMB** ]  and caspase 3  [ **CASP3** ] .  Ref: 11866458 Biochem Biophys Res Commun, 2002
- Second, the delivery system mediated delivery of several caspases caspase 3  [ **CASP3** ] , caspase 8, and granzyme B  [ **GZMB** ]  into cultured cell lines and primary cells triggering apoptosis.  Ref: 11447231 J Biol Chem, 2001
- DFF45 ICAD cleavage occurs less efficiently in cells that lack caspase 3  [ **CASP3** ]  activity, suggesting that the caspases normally amplify the  **GzmB**  death signal.  Ref: 10894162 Immunity, 2000
- Caspase activation is not required for cytochrome c release, an activity that correlates with cleavage and activation of Bid, which we have found to be cleaved more readily by granzyme B  [ **GZMB** ]  than either caspase 3  [ **CASP3** ]  or caspase 8.  Ref: 11278459 J Biol Chem, 2001
- Treatment of HL 60 cells with lactacystin, a selective inhibitor of the proteasome, exponentially increased caspase 3  [ **CASP3** ]  like hydrolytic activity and induced apoptosis but had little or no effect on the activity of upstream caspase 8, caspase 9, or granzyme B  [ **GZMB** ] .  Ref: 12869638 Mol Pharmacol, 2003
